# Sheeppox and Goatpox: Molecular Epidemiology, Phylogeny and Transmission Patterns

**DOI:** 10.3390/v18080849

**Published:** 2026-08-03

**Authors:** Alexander Sprygin, Fedor Korennoy, Rajabmurod Atovullozoda, Shawn Babiuk, Oksana Vernygora, Oliver Lung, Mohammad Abed Alhussen, Sulaimon Nazrullozoda, Natalia Yarygina, Nikita Tenitilov, Olga Byadovskaya, Ilya Chvala

**Affiliations:** 1 Federal Centre for Animal Health (FGBI ARRIAH), ul. Gvardeyskaya 6, Yur’evets, Vladimir 600901, Russia; korennoy@arriah.ru (F.K.);; 2 Institute of Veterinary Medicine of the Tajik Academy of Agricultural Sciences, 43 A. Kahorov, St., Dushanbe 734005, Tajikistan; 3 National Centre for Foreign Animal Disease, Canadian Food Inspection Agency, 1015 Arlington Street, Winnipeg, MB R3E 3R2, Canada; shawn.babiuk@inspection.gc.ca (S.B.); oksana.vernygora@inspection.gc.ca (O.V.); oliver.lung@inspection.gc.ca (O.L.)

**Keywords:** sheeppox and goatpox, spatiotemporal, transmission, phylogeny, recombination

## Abstract

Sheep- and goatpox are highly contagious, transboundary viral diseases caused by capripoxviruses (CaPV), severely impacting small ruminant production and resulting in significant economic losses. Our study aimed to analyze the spatiotemporal SGP distribution using the available genome sequence data and to evaluate the recombination occurrence. For this, the WAHIS, WOAH and FAO databases were utilized for the epidemiological analysis of SGP outbreaks. The cross-correlation was calculated to assess the impact of massive animal movements associated with the Islamic holiday of Eid al-Adha on the SGP epizootic situation, and phylodynamic and phylogeographic analysis, as well as a recombination analysis were performed. A total of 1629 SGP outbreaks were reported to WAHIS during 2010–2024, with the majority occurring in Mongolia, the Balkan countries, Russia and the Middle East. Over 60% of all SGP outbreaks were associated with croplands or grasslands, with the highest proportion corresponding to animal densities of 10–50 head/km^2^. Statistically significant positive cross-correlation (*p* < 0.05) was identified between the month of the Eid al-Adha celebration and the number of SGP outbreaks in Russia, Mongolia, Bulgaria and Tajikistan, while in Greece and China no significant correlation was found. The inferred goat pox virus (GTPV) transmission pathways from China to Vietnam and from India to Bangladesh; for the sheeppox virus SPPV, the routes between Kazakhstan and Russia, Kazakhstan and India, as well as between Russia and China, had the strongest Bayes factor support. Intra-specific recombination events were not detected for the SSPV and GTPV datasets. However, inter-specific CaPV recombination analysis identified a single recombination event in GTPV. Therefore, the use of molecular epidemiological tools, along with the time-calibrated phylodynamic and phylogeographic analyses, has significant applications in the local and international surveillance of the occurrence of SGP outbreaks and for identification of potential recombination events.

## 1. Introduction

The *Capripoxvirus* genus, belonging to the Poxviridae family, includes the species *Capripoxvirus sheeppox* (sheeppox virus, SPV), *Capripoxvirus goatpox* (goatpox virus, GPV) and *Capripoxvirus lumpyskinpox* (lumpy skin disease virus, LSDV) [1,2]. The host range of capripoxviruses (CaPVs) is host-restricted; lumpy skin disease (LSD) primarily affects cattle and Asian water buffalo, whereas SPPV and GTPV affecting sheep and goats, respectively, with some isolates are able to infect both species [3,4,5]. Furthermore, CaPV infection causes significant economic losses in livestock production in regions where these diseases are endemic and classified as notifiable diseases by the World Organization for Animal Health (WOAH) [4,6,7]. The emergence of these diseases poses a substantial threat to the economies of the previously disease-free countries due to the imposition of trade restrictions as well as substantial control and eradication costs [4,8,9]. Sheeppox and goatpox (SGP) are diseases characterized by fever and skin lesions, with a high morbidity (70–90%) and mortality up to 50 % (up to 100% in young stock) rates [5,8,10].

The geographic range of SGP in small ruminants has exhibited relative stability, remaining endemic in northern and central Africa, the Middle East, and several parts of Asia, including India and China [7,11]. However, a comparative analysis of their geographic ranges and evolutionary histories reveals differences, including the absence of GTPV in Morocco and Russia [12].

The SGP outbreaks caused significant concern in regions bordering the Russian Federation, where the virus is endemic in several neighboring areas. GTPV outbreaks were reported in Vietnam (2005, 2014) [13], Mongolia (2008, 2009) [14] and Chinese Taipei (2008, 2010) [15]. SPPV outbreaks have been documented in Mongolia (2006–2007, 2025) [14], Spain (2022–2023) [16], Greece (2013, 2022, 2023, 2024) [17], Bulgaria (2013, 2022, 2023, 2024, 2025), Romania (2025) and Serbia (2025) [17].

The transmission of the SGP virus (SGPV) primarily occurs through close and direct contact with the infected animals via respiratory droplets or skin abrasions in crowded settings such as markets or pastures. The indirect transmission can occur through contact with the contaminated equipment or bedding on infected premises, given that CaPVs exhibit stability in dry environments [2,8,18]. Furthermore, the spread of SGP is attributable to the legal and illegal trade of animals and their products, as well as virus-contaminated fomites, demonstrating their transboundary nature [7,19]. Notably, the precise cause of the SPPV outbreak in Spain remains unidentified [16].

The control of SGP outbreaks can be accomplished through the implementation of movement restrictions and stamping out [2]. In endemic regions, several live attenuated vaccines are employed for disease control; however, insufficient vaccination coverage prevents eradication, thereby enabling the ongoing circulation of SGP [8,10].

CaPVs demonstrate over 96% genomic similarity across their entire genome lengths and are generally genetically stable viruses [20]; however, several distinct recombinant lumpy skin disease viruses have been recently identified in Russia, and one of these recombinant LSDV has established itself in many countries in Asia [21]. The possibility of recombination between the SPPV and GTPV was previously suggested [20,22].

The SGPV genomes are approximately 150 kbp, contain 147–156 putative genes and are phylogenetically similar. Generally, SGP displays a host preference for either sheep or goats with some isolates in the Middle East able to infect both species. Different genes, such as P32, RPO30 and GPCR, are used to identify SPPV or GTPV [20,23]. Nevertheless, the use of partial sequence data based on specific genes to characterize CaPVs does not provide the complete picture compared to whole genome sequencing (WGS), primarily given their high genomic homology. The transmission and spread of LSDV has been previously demonstrated using spatiotemporal epidemiology [24,25,26].

The present study aimed to assess the geographic and temporal distribution of sheeppox and goatpox using the available genome sequence data and to evaluate the occurrence of recombination.

## 2. Materials and Methods

### 2.1. Sheeppox and Goatpox Data

The study employed three complementary data sources on the global SGP outbreaks from 2010 to 2024:

The World Animal Health Information System (WAHIS), managed by the World Organization for Animal Health (WOAH) (https://wahis.woah.org/#/event-management (accessed on 20 July 2026)). This dataset encompasses geographic coordinates and outbreak start dates, rendering it conducive to for spatial and temporal analysis. In this context, an outbreak is defined as a case of SGP occurrence in a susceptible population, localized by geographic coordinates and a specific date.

WOAH Semi-Annual and Annual Reports (https://wahis.woah.org/#/smr-management, https://wahis.woah.org/#/annual-report (accessed on 20 July 2026)). These reports provide more complete coverage of countries where SGP was reported and used to verify the overall morbidity picture. However, due to the lack of precise geolocation data, this dataset was not used for spatial analysis.

The FAO EMPRES-i database (Emergency Prevention System Global Animal Disease Information System) (https://empres-i.apps.fao.org/diseases (accessed on 20 July 2026)). This information source is considered a robust global tool providing comprehensive data on the geographical and temporal characteristics of recorded outbreaks; however, it has significant limitations regarding data coverage, including data gaps and incomplete reporting by countries, as well as inconsistencies in case definitions.

Additionally, we employed data on SGP outbreaks in Tajikistan (2018–2024), provided by Institute of Veterinary Medicine of the Tajik Academy of Agricultural Sciences. These data, collected at the national level, do not include numbers of either susceptible or infected animals, but do include the date of disease onset and were therefore used only for time series analysis.

### 2.2. Epidemiological Analysis

The WAHIS data were utilized to calculate key epidemiological indicators for each documented outbreak:

— Morbidity is defined as the ratio of the number of sick animals to the total number of susceptible animals in the population at risk within a specific time frame.
— Case fatality rate is calculated as the ratio of the number of dead animals to the number of sick animals.
— Mortality is expressed as the ratio of the number of dead animals to the total number of susceptible animals.

To analyze of the influence of population size, all affected farms/holdings were categorized by the number of susceptible animals as follows: small (≤100 heads), medium (101–1000 heads), and large (>1000 heads). The outbreak seasonality was visualized using a radar chart, displaying the distribution of cases by month.

Cross-correlation analysis of time was applied to evaluate the hypothesis regarding the impact of animal movements associated with the Islamic holiday of Eid al-Adha on the SGP epizootic situation [27,28]. The initial hypothesis suggested that the intensification of trade and the importation of animals from endemic regions during the pre-holiday period could result in a subsequent increase in the number of recorded outbreaks [29].

To conduct the analysis, two time series were constructed:

— A predictor series—the months of the Eid al-Adha celebration for the period 2013–2024, where a value of “1” means the month corresponding to the month in which the holiday occurred.
— A resulting series—the monthly number of recorded SGP outbreaks in the territory of an analyzed country over the same period.

The calculation of the cross-correlation function (CCF) enables the evaluation of the strength and direction of the relationship between two time series at various time shifts (lags). In our case, positive lag values (k > 0) indicate that changes in the predictor series (the month of the holiday) preceded changes in the resulting series (the number of outbreaks). The presence of a statistically significant peak in cross-correlation at a positive lag (for example, one to two months) could indicate that the importation of animals for the holiday, followed by the development of the epizootic process (considering the incubation period and the time required to detect outbreaks), indeed influences the observed morbidity dynamics.

The analysis was conducted for the Russian Federation and several other countries, for which data on the monthly dynamics of SGP outbreaks over a comparable period (2013–2024) were available, included China, Mongolia, Bulgaria, Greece and Tajikistan. Cross-correlation was calculated using the cross-correlation function (CCF) in R programming environment.

### 2.3. Spatial Analysis

To identify the predominant geographic and demographic confinement of SGP outbreaks, a spatial analysis, using only the WAHIS point data, was carried out in relation to the following: eco-geographic zones (global ecosystems); land cover types; and average density of small cattle in the world.

Global ecosystem data were obtained from the Esri World Terrestrial Ecosystems dataset (250 m resolution), consisting of 431 unique ecosystem types by combining climate regions, land cover types, and landform classes [30,31].

The MODIS/Terra Land Cover Types dataset (MCD12Q1) (https://developers.google.com/earth-engine/datasets/catalog/MODIS_061_MCD12Q1; accessed on 20 July 2026) was utilized to provide global land cover at a resolution of 500 m, featuring 17 distinct classifications. The small ruminant density data (sheep and goats) were obtained by merging the FAO Gridded Livestock of the World (GLW) datasets on sheep and goat density, which provides global coverage at a resolution of 10 km (https://www.fao.org/livestock-systems/global-distributions/en/; accessed on 20 July 2026).

To determine the association of outbreaks with categories of each layer, a 2.5 km correction zone around each outbreak point was created. This radius was selected to compensate for potential geolocation inaccuracies and to align the scale with the analyzed raster data. For each zone, the predominant category value (Majority) within the zone was determined using the Zonal Statistics GIS tool. This value was then considered the characteristic associated with the outbreak. Furthermore, the proportion of outbreaks in each category was calculated for each factor.

Mapping and spatial analysis were performed using the geographic information system ArcGIS for Desktop 10.8.2 (Esri, Redlands, CA, USA).

### 2.4. Phylogenetic and Phylogeographic Analysis

For the phylogenetic analysis, all complete and nearly complete (sequence length > 100,000 nt) genome sequences of the three capripoxvirus species (n = 305) were downloaded from the NCBI Virus portal (accessed on 28 November 2025). Two additional not publicly available genome sequences were added to the publicly available records. The final dataset comprised a total of 307 sequence records (GTPV 15; SPPV 36; LSDV 256). Multiple sequence alignment was performed using MAFFT v7.511 under the default settings. The maximum likelihood phylogenetic analysis was performed using IQ-TREE v3.01 [32]. The best-fit model of sequence evolution was selected based on the Bayesian information criterion (BIC) score [33] calculated by ModelFinder [34]. K3Pu + F + R7 was selected as the best-fitting model for the analysis. Node support was estimated by ultrafast bootstrap [35] and the SH-aLRT test [36] with 1000 replicates each.

Time-calibrated phylodynamic and phylogeographic analysis was performed for GTPV (n = 13) and SPPV (n = 30) sequence records that had collection date and location information available. Phylodynamic and geographical reconstructions were performed using BEAST v1.10.4 [37] with discrete trait reconstruction under the asymmetric substitution model and the Bayesian Stochastic Search Variable Selection procedure enabled. Time-calibrated phylogenetic analysis was performed using a coalescent model with constant population size [38,39] and a general time-reversible substitution model with the across-site rate heterogeneity sampled from a gamma distribution with four discrete categories, and relaxed log-normal molecular clock. Four independent analyses were run for 200 million generations each and parameter values were recorded every 5000 generations. Stationarity, convergence, and the burn-in fraction across independent runs were assessed in Tracer v1.7 [40]. The tree and log files were combined using the LogCombiner module of the BEAST software, and the final maximum clade credibility tree with median node heights was produced using TreeAnnotator v1.7.5 [41]. Phylogeographic reconstruction and visualization were performed using SpreaD3 v0.9.6 [42]. The final results visualization was performed using FigTree v.1.4.4 (https://github.com/rambaut/figtree (accessed on 20 July 2026)) and Inkscape v1.0.2-2 (https://inkscape.org/ (accessed on 20 July 2026)).

### 2.5. Recombination Analysis

A series of inter- and intra-specific recombination analysis were performed using the RDP5 v.5.30 [43] under the default settings and with the *p*-value cut-off set to 0.01. For the intra-specific recombination analysis, datasets comprising GTPV and SPPV sequences only were analyzed separately; for the interspecific recombination analysis, the complete dataset comprising sequence records of all three species was used (n = 307). The initial identification of potential recombination events and breakpoints was performed using five detection methods (RDP, GENECONV, 3Seq, MaxChi, and Chimaera). The identified recombination events were then checked with BootScan and SiScan [44]. Recombination events that were detected by at least five out of seven methods were selected as plausible.

## 3. Results

### 3.1. SGP Outbreaks Worldwide from 2010 to 2024

**Figure 1:**
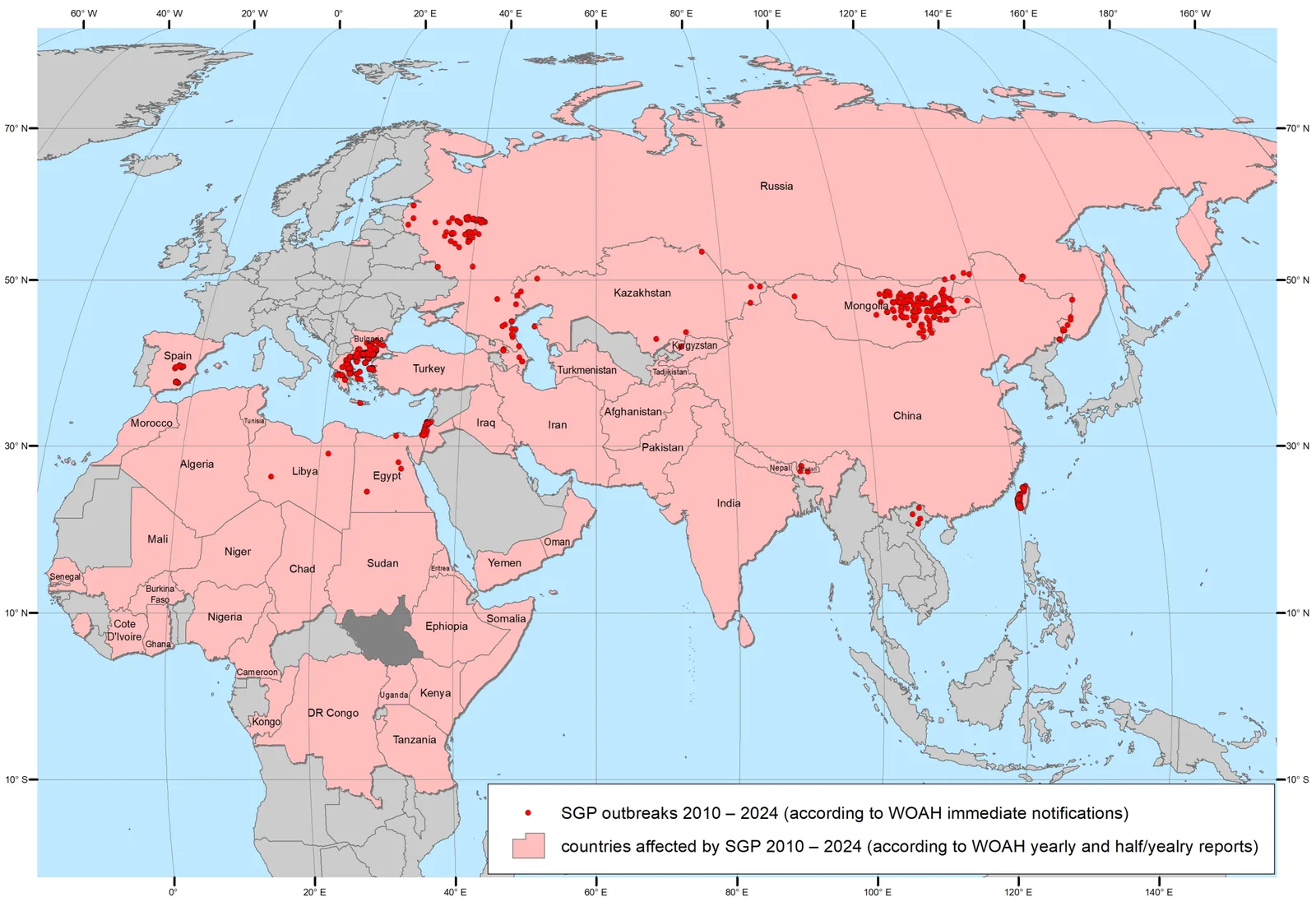
Reported SGP outbreaks during 2010–2024 according to WOAH WAHIS.

From 2010 to 2024, a total of 1629 SGP outbreaks were officially notified to WAHIS. Concurrently, 55 countries across Eurasia and Africa reported the disease (Figure 1). The majority of the reported outbreaks occurred in countries including Mongolia, the Balkan countries, Russia and the Middle East.

### 3.2. Epidemiological Analysis Results

The data indicated that goats, sheep and mixed herds were affected in 393 (24%), 1046 (64%), and 190 (12%) of the 1629 recorded outbreaks, respectively. In 154 of the total number of outbreaks, the number of susceptible animals was not provided, whereas in 121 cases, it was recorded as zero. The morbidity and mortality proportions were calculated for the remaining outbreaks herd-level. The results are presented in Table 1.

The epidemiological data presented in the table demonstrate a decrease in both the number of recorded SGP outbreaks and the morbidity when compared with the increase in the number of susceptible animals. The highest frequency of outbreaks (n = 686) and the highest mean morbidity (21.0%) was observed in small-scale holdings with less than 100 head. As the number of susceptible animals increased, the morbidity rate exhibited a marked decline, dropping to 6.6% in medium-sized flocks and reaching a minimum of 3.1% in large-scale operations (more than 1000 head). Interestingly, mortality did not mirror the steep downward trend seen for morbidity. The overall decrease was only slight (from 2.5% to 1.8%), indicating that, irrespective of flock size, once the pathogen becomes established, the likelihood of death remains appreciably similar across all production scales.

**Figure 2:**
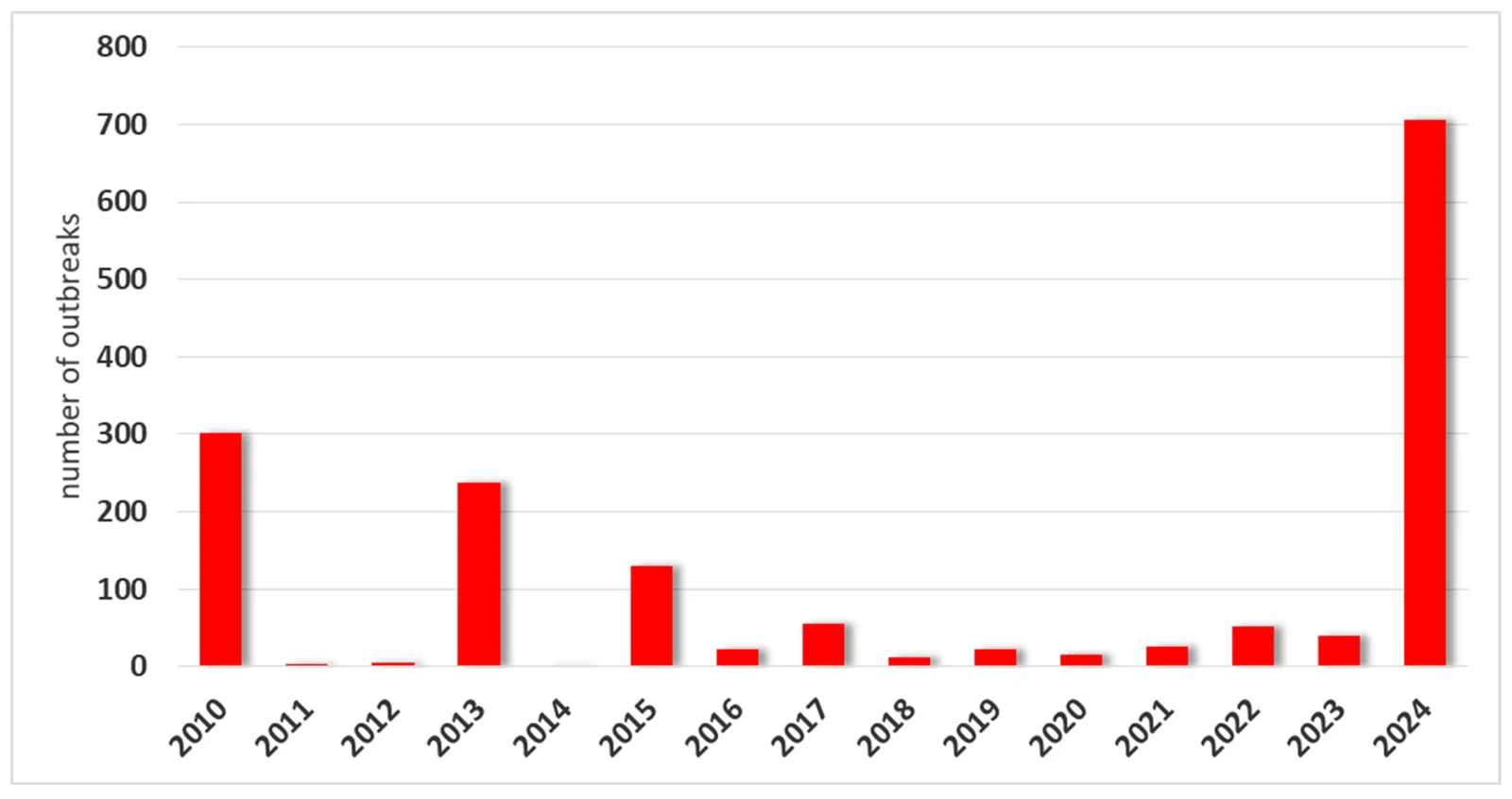
Annual distribution of the number of SGP outbreaks reported globally from 2010 to 2024 according to WOAH WAHIS.

The distribution of the number of outbreaks worldwide by year (Figure 2) indicates that the annual reported SGP outbreaks during 2010–2024 exhibited instability. In the period between 2011 and 2023, the number of notified outbreaks remained at relatively low levels, with minor increases observed in 2010, 2013 and 2015 (301, 237 and 129, respectively). During the 2016–2023 period, the annual number of outbreaks remained below 100, indicating a period of relative stability or localized endemicity. However, a remarkable increase in the number of reported SGP outbreaks was observed in 2024, reaching 706. This represents a more than ten-fold increase compared to the average of the preceding decade, suggesting a significant shift in the transboundary spread of the virus or enhanced surveillance and reporting mechanisms within the affected regions.

**Figure 3:**
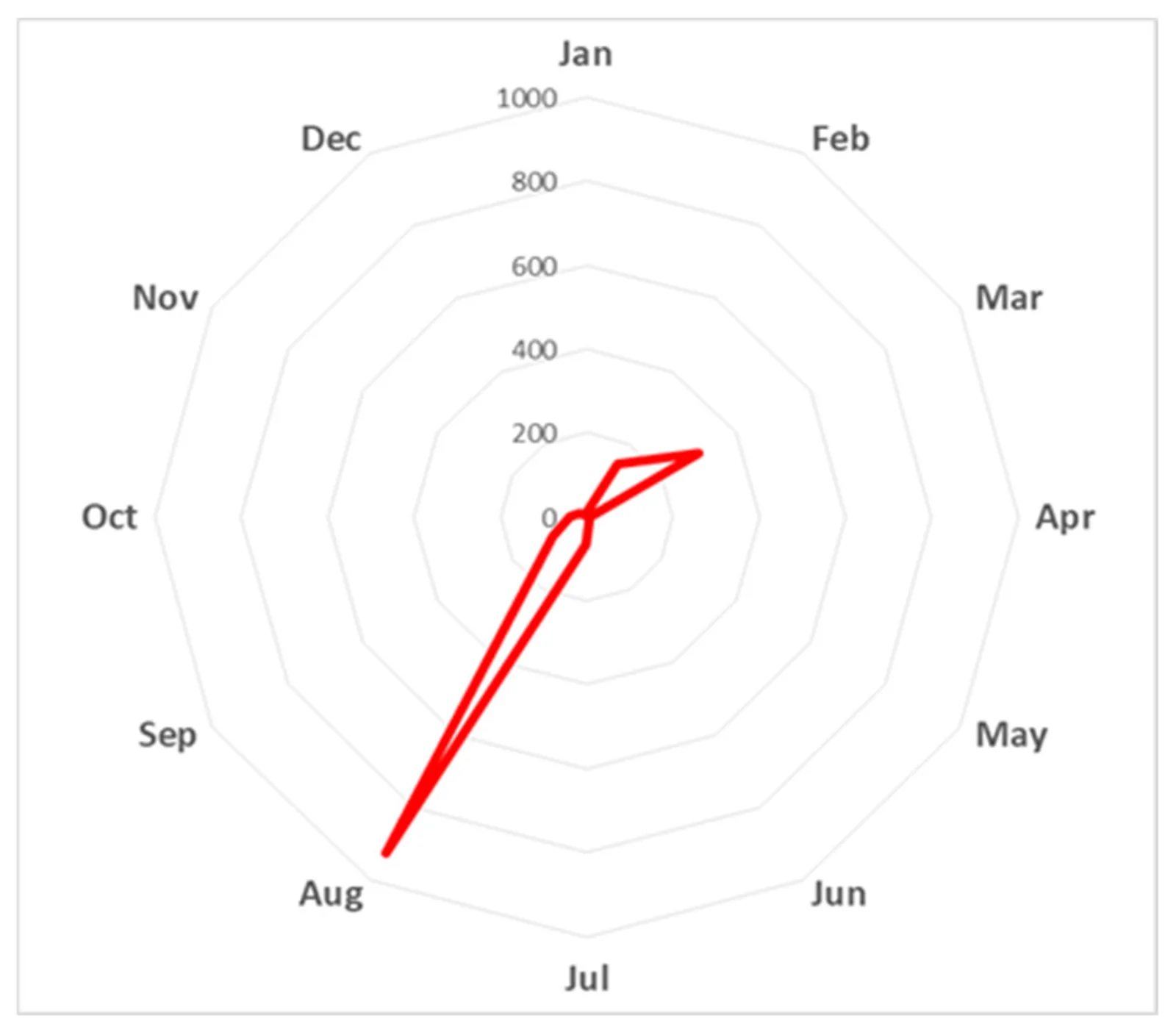
Radar chart illustrating the monthly seasonality of SGP outbreaks during 2010–2024. The radial axis represents the total number of outbreaks.

Seasonality analysis of the SGP outbreaks from 2010 to 2024 (Figure 3) reveals a distinct seasonal pattern (Figure 3), characterized by a surge during the late summer months, where a primary sharp peak is observed in August, reaching the highest recorded frequency of approximately 800 outbreaks. A secondary, less pronounced, increase is noted in the early spring months of February and March.

**Figure 4:**
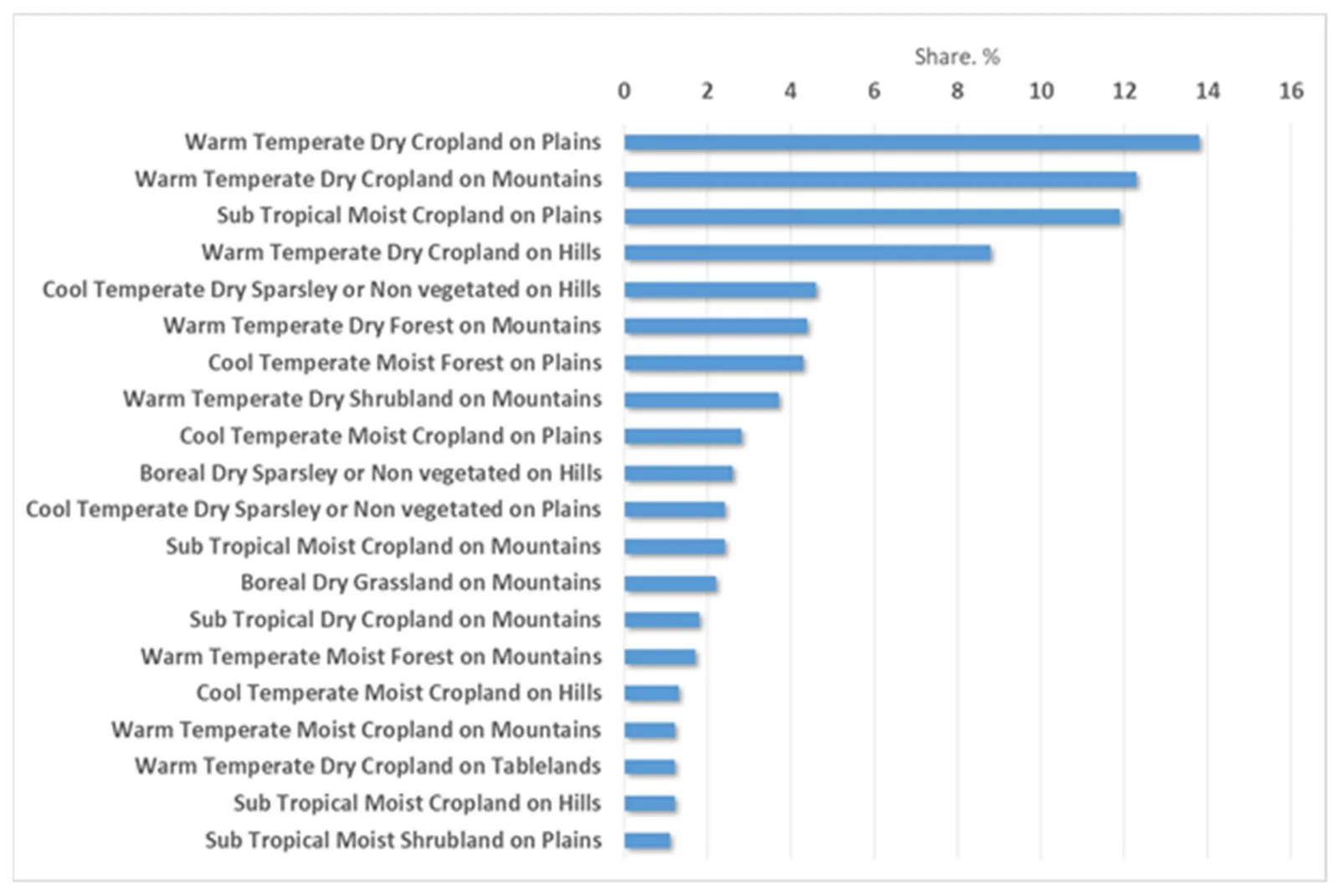
Contribution of different ecosystem types to the SGP outbreaks (sourced from FAO EMPRES-i) during 2010–2024; only ecosystems representing >1% of the total recorded outbreaks are presented herein.

The analysis of the ecological types and their relation to the SGP outbreaks during 2010–2024 indicates that over 40% of all recorded SGP outbreaks occurred in four ecosystem types, with warm temperate dry cropland on plains being the most frequent, accounting for approximately 13.8% of the total (Figure 4). The subsequent categories were warm temperate dry cropland on mountains and subtropical moist cropland on plains (12.3% and 11.9%, respectively).

Interestingly, while dry ecosystems dominate the high-frequency categories, “moist” subtropical and temperate forests and croplands also contributed significantly to outbreak occurrences, ranging from 1% to 12%. Conversely, more severe environments, such as boreal dry grasslands or shrublands, represented, lower proportions, typically around 2–3%.

**Figure 5:**
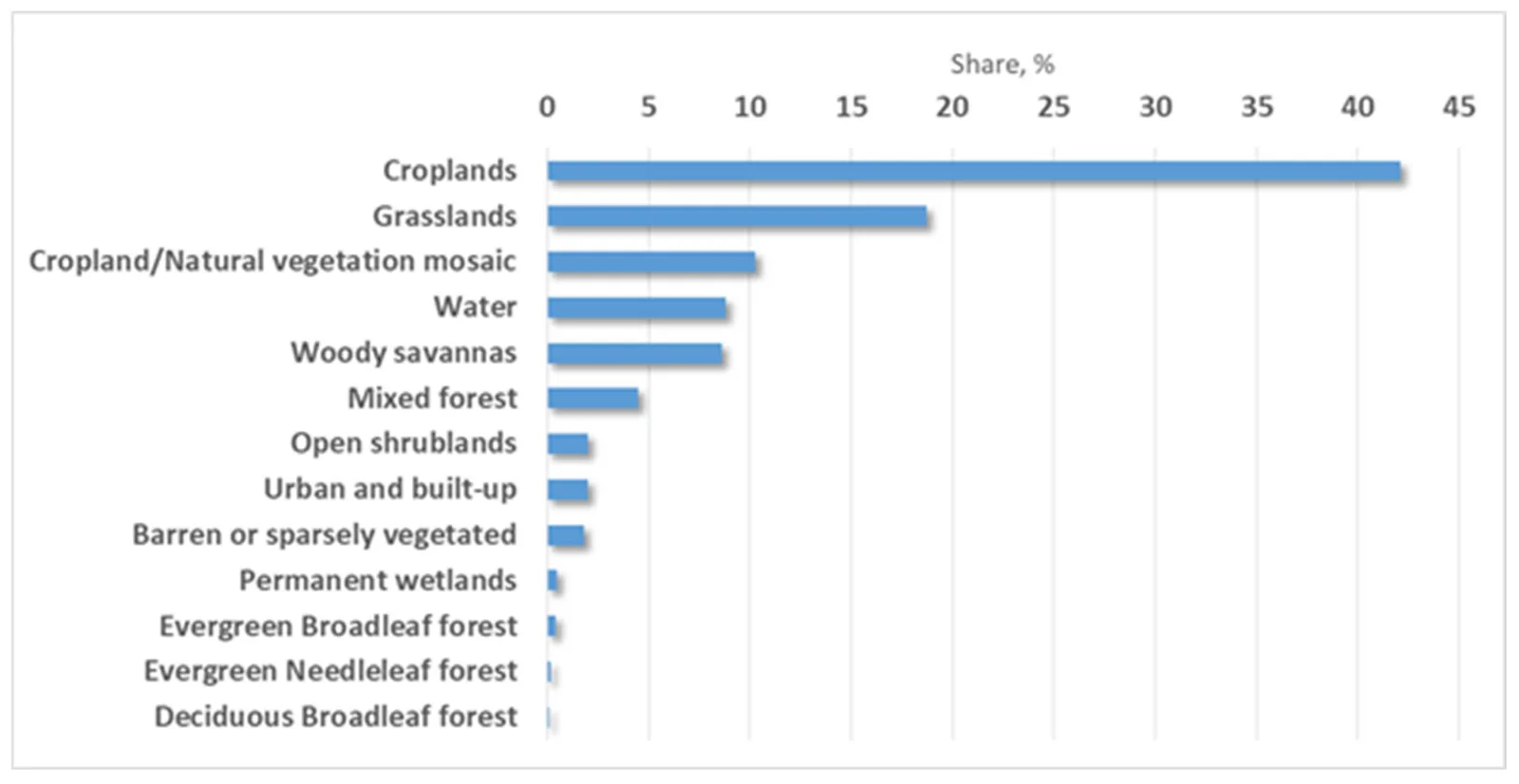
Proportional contribution of various land cover types to SGP outbreaks (sourced from FAO EMPRES-i) recorded between 2010 and 2024.

The analysis of the various land cover types reveals that the majority of SGP outbreaks are associated with landscapes containing croplands, which emerged as the primary land cover category, accounting for over 42% of all reported SGP outbreaks (Figure 5). This finding is consistent with the results of the ecosystem-type analysis (Figure 4). The second-largest category, comprising approximately 18% of the total share, corresponded to the grasslands.

**Figure 6:**
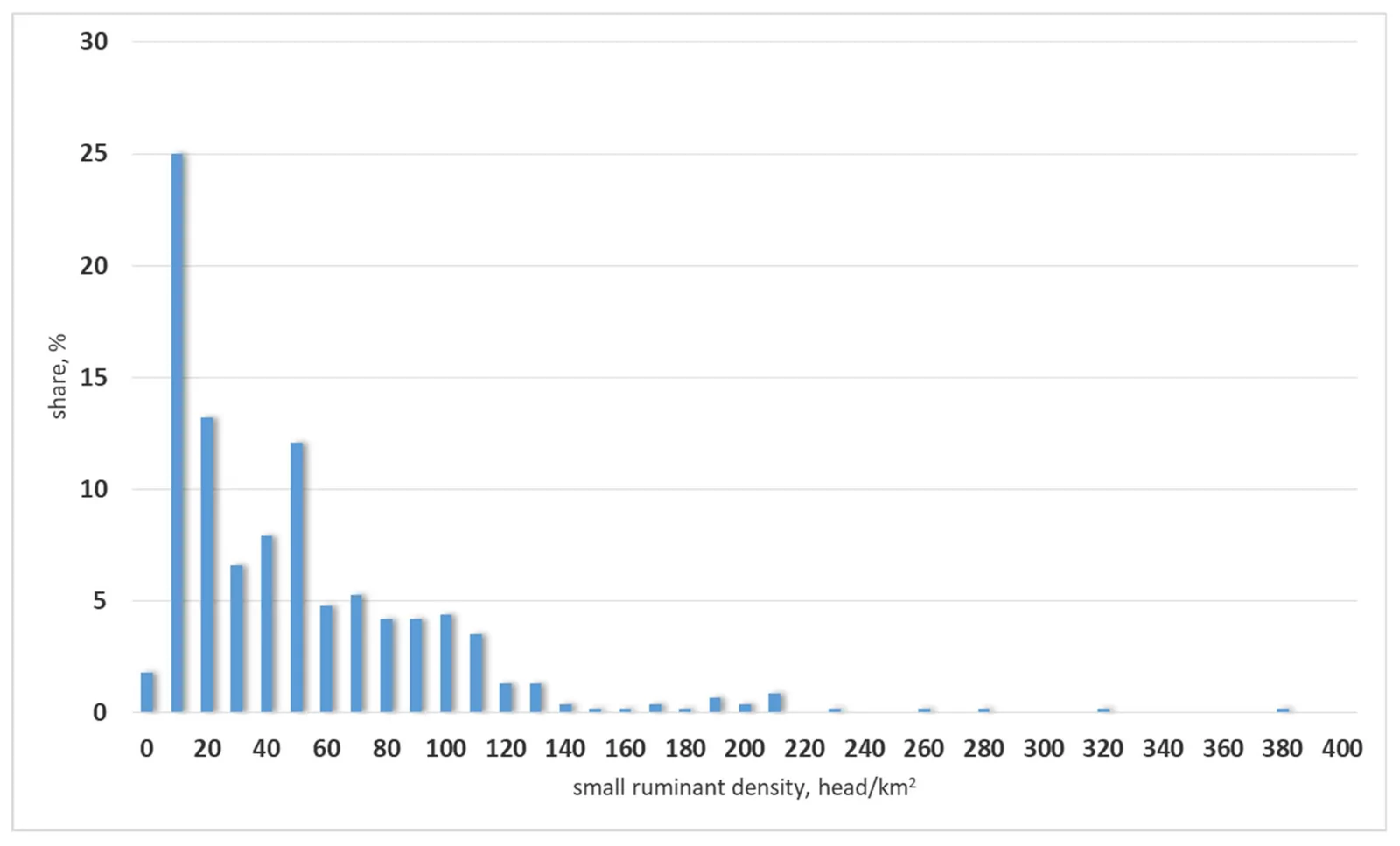
Distribution of small ruminant population density (head/km^2^) at the locations of SGP outbreaks reported between 2010 and 2024.

The analysis of the small ruminant population density at SGP outbreak sites (2010–2024) indicates that most outbreaks are clustered in areas with average density of small ruminants less than 140 head/km^2^. The highest incidence of outbreaks (25%) was observed in regions with a density of approximately 10–20 head/km^2^. A notable proportion of SGP outbreaks persisted as density increased up to 50 head/km^2^, after which a precipitous decline in frequency of outbreaks was observed (Figure 6).

### 3.3. Results of the Cross-Correlation Analysis of Eid al-Adha Festivals and SGP Outbreaks During 2013–2024

**Figure 7:**
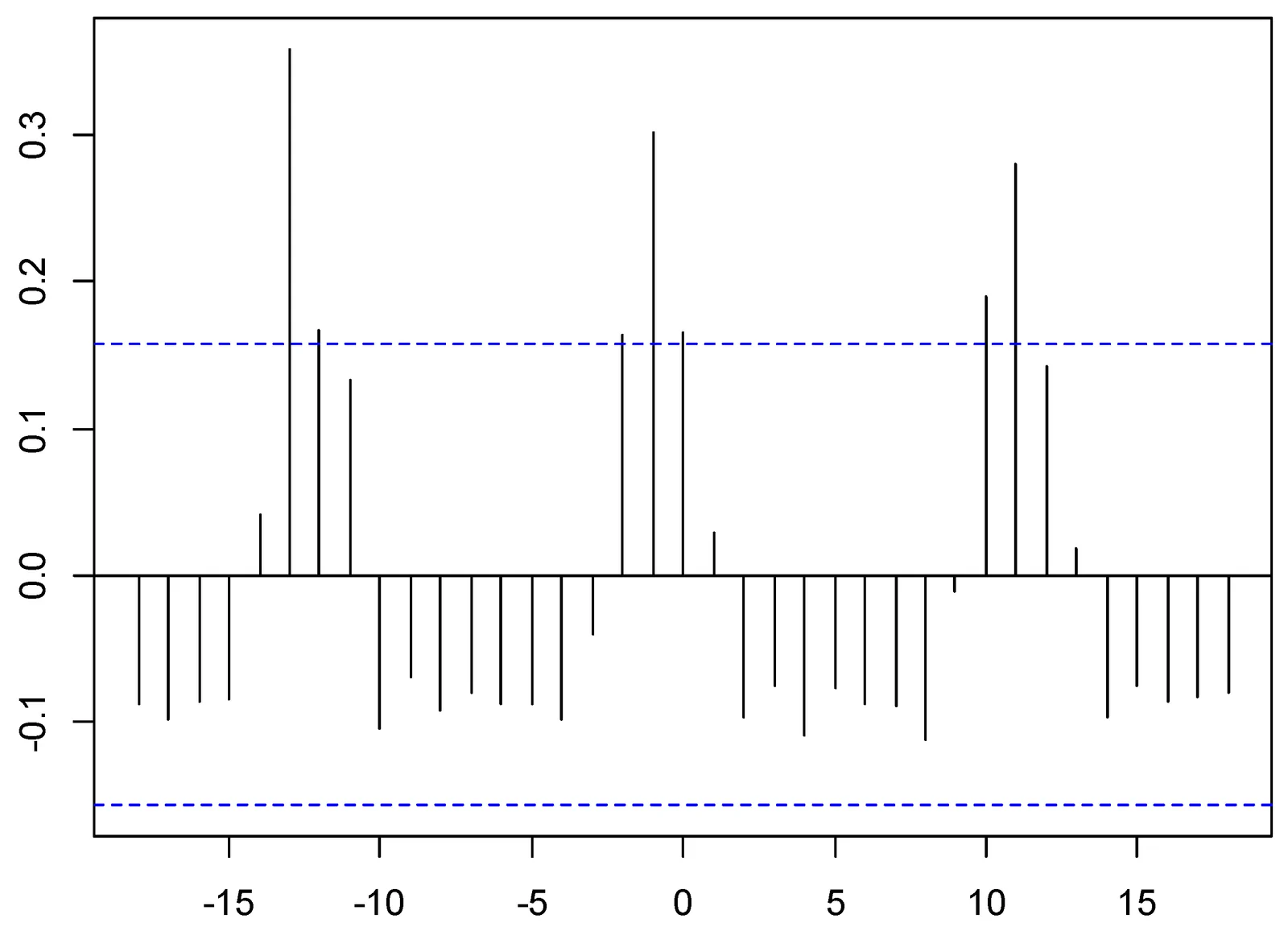
Cross-correlation function (CCF) between time series representing Eid al-Adha month and monthly number of SGP outbreaks in Russia in 2013–2024. In Figure 7, Figure 8, Figure 9, Figure 10 and Figure 11, a horizontal axis represents time lag in months; a vertical axis demonstrates CCF as a correlation coefficient. Dashed lines are employed to denote a threshold, beyond which CCF values demonstrate statistical significance.

**Figure 8:**
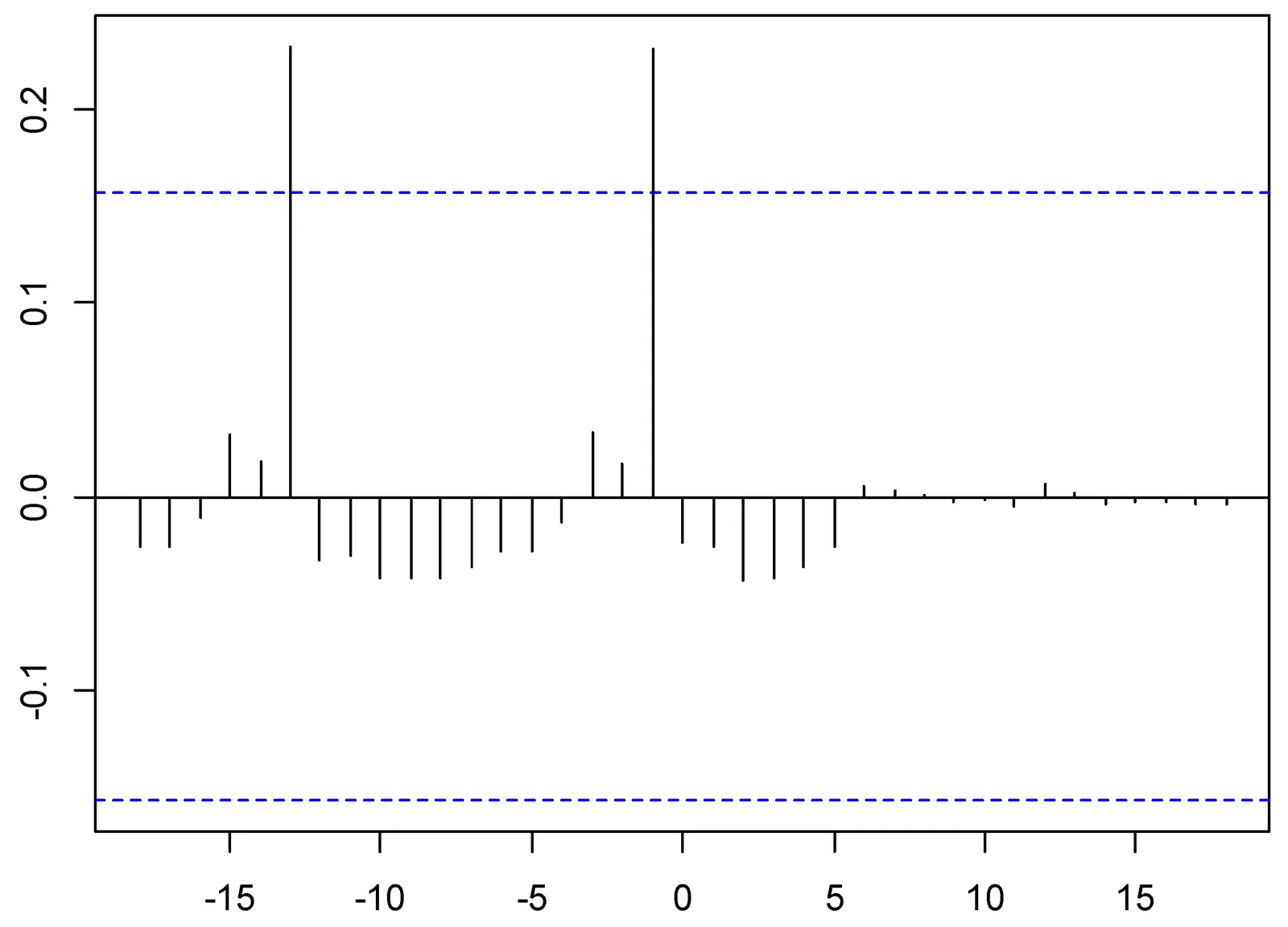
Cross-correlation function (CCF) between time series representing Eid al-Adha month and monthly number of SGP outbreaks in Bulgaria in 2013–2024.

**Figure 9:**
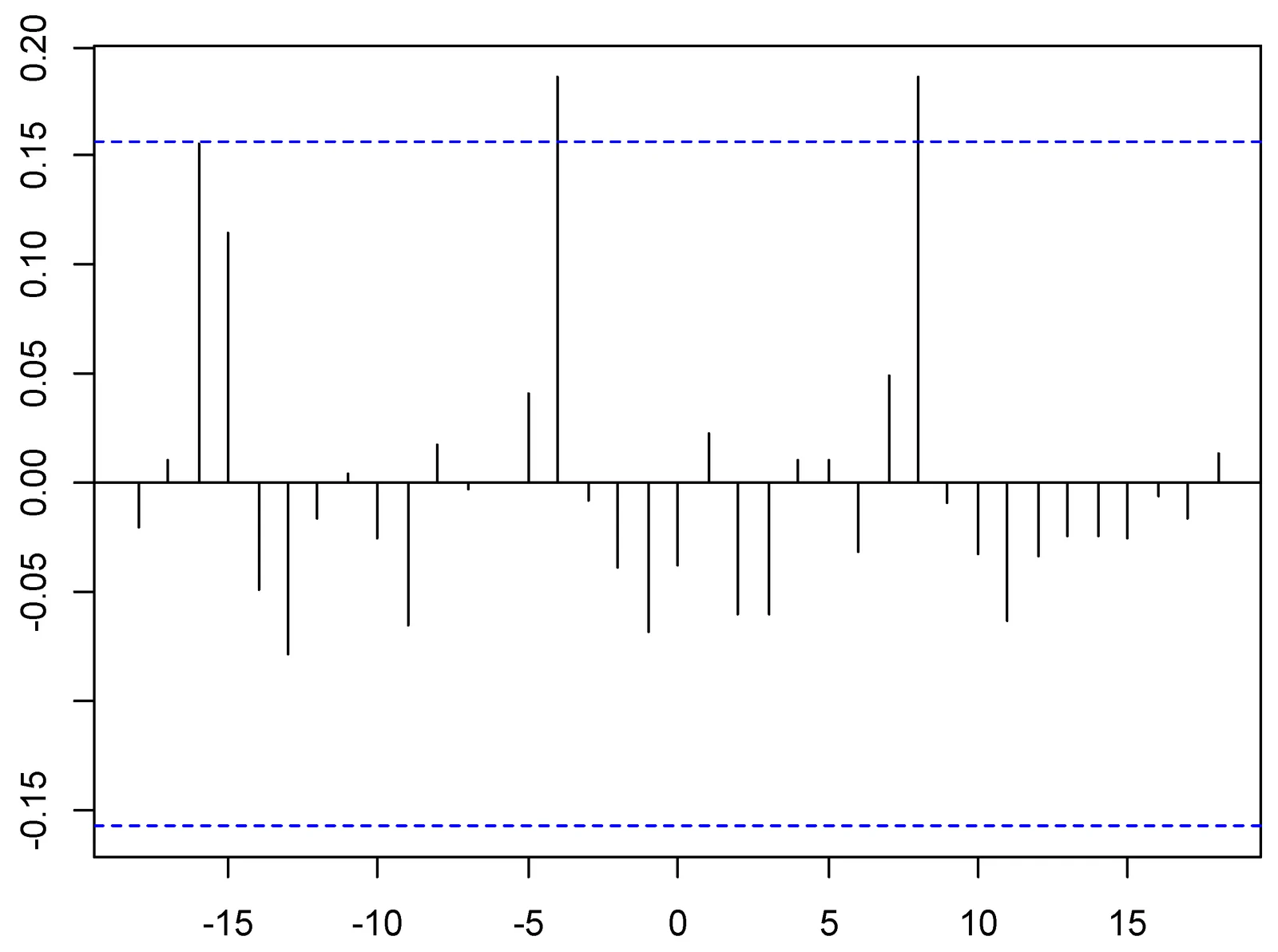
Cross-correlation function (CCF) between time series representing Eid al-Adha month and monthly number of SGP outbreaks in Mongolia in 2013–2024.

**Figure 10:**
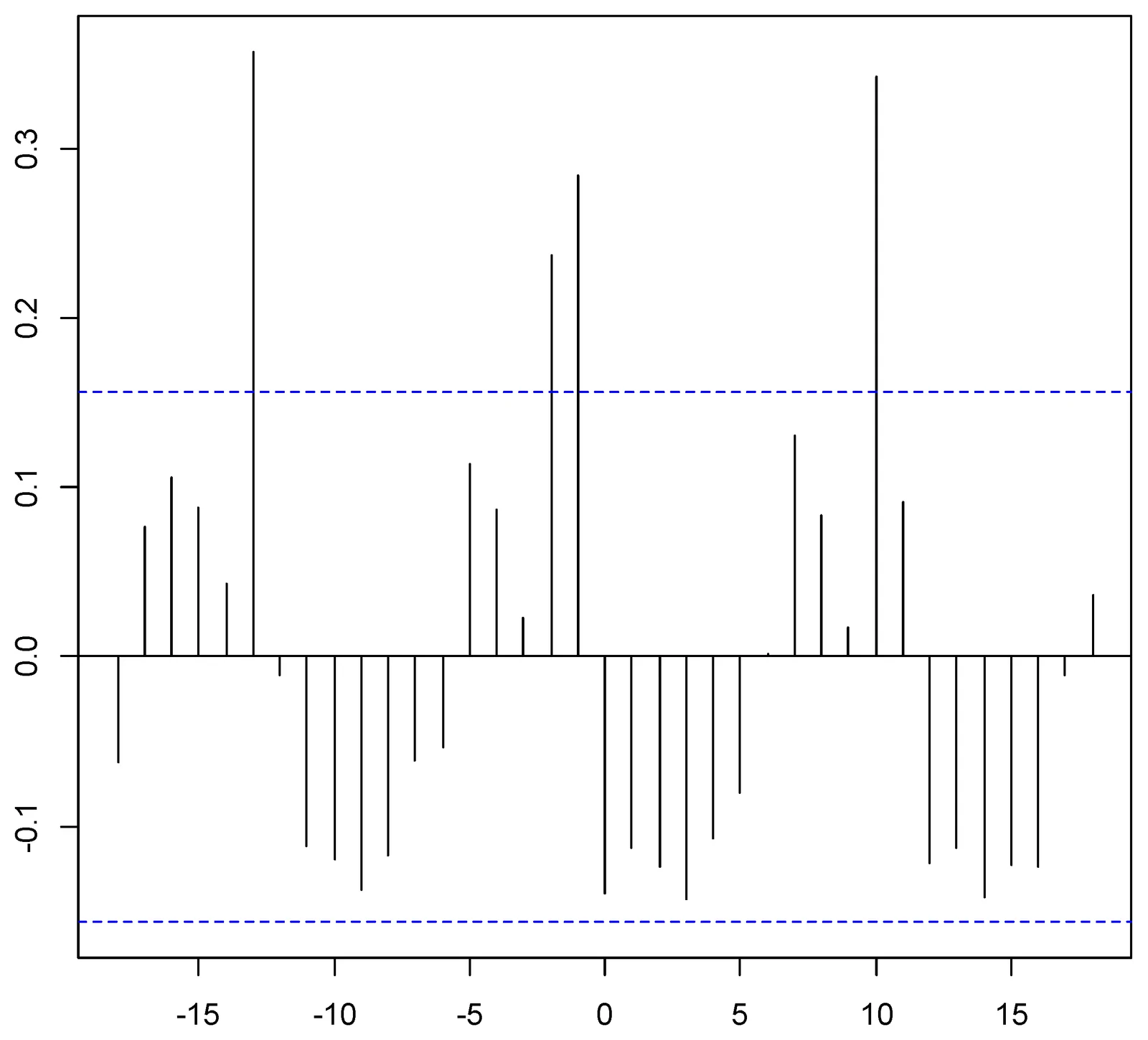
Cross-correlation function (CCF) between time series representing Eid al-Adha month and monthly number of SGP outbreaks in Tajikistan in 2013–2024.

The data revealed statistically significant positive cross-correlation was found between the binary predictor (the month of Eid al-Adha celebration) and the number of SGP outbreaks at a time lag of one month (*p* < 0.05) in the Russian Federation and Bulgaria during 2013–2024 (Figure 7 and Figure 8). A similar pattern was identified in Mongolia with a time lag of 3–4 months (Figure 9), and in Tajikistan with a time lag of one month (Figure 10).

**Figure 11:**
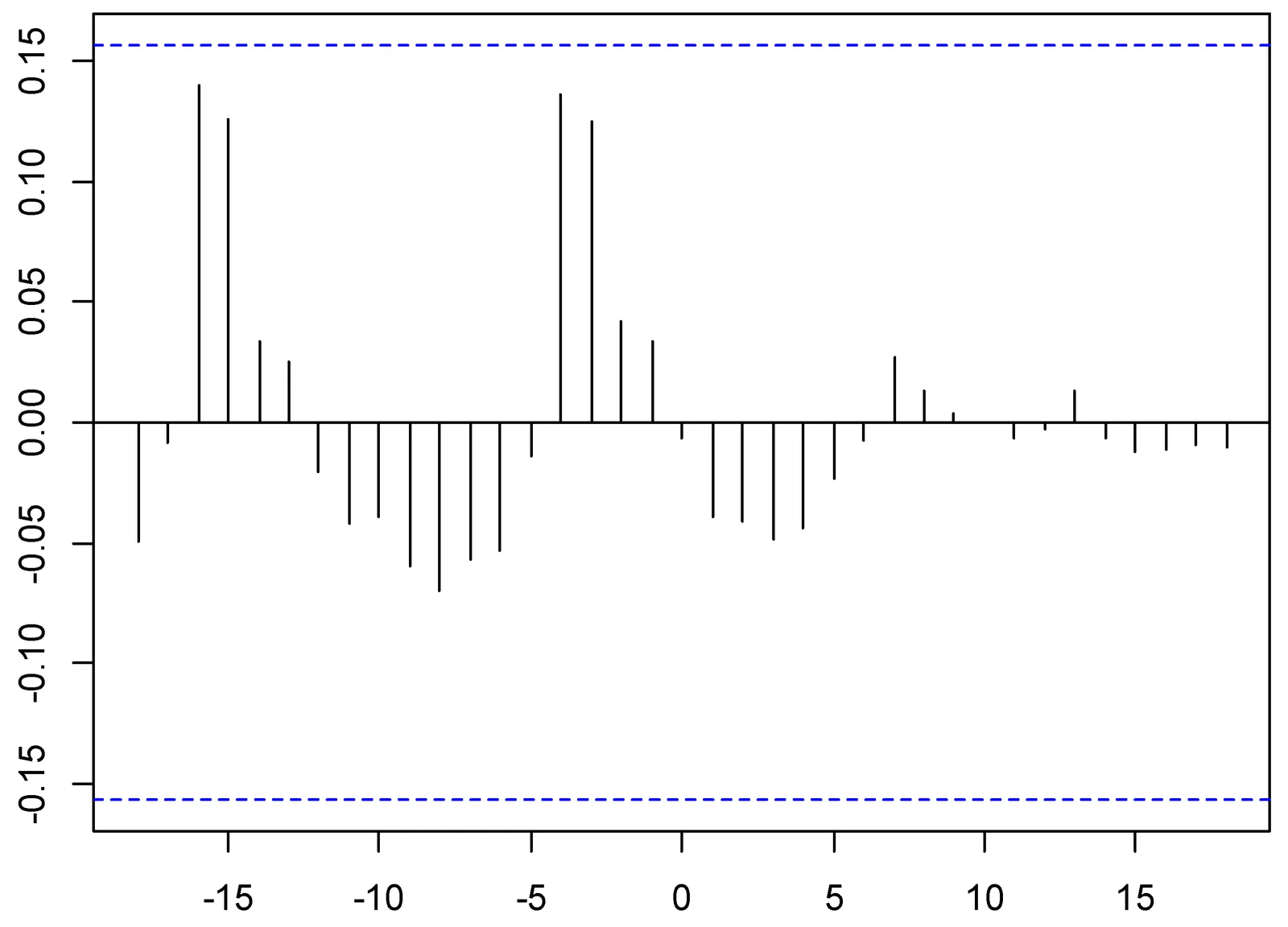
Cross-correlation function (CCF) between time series representing Eid al-Adha month and monthly number of SGP outbreaks in Greece in 2013–2024.

**Figure 12:**
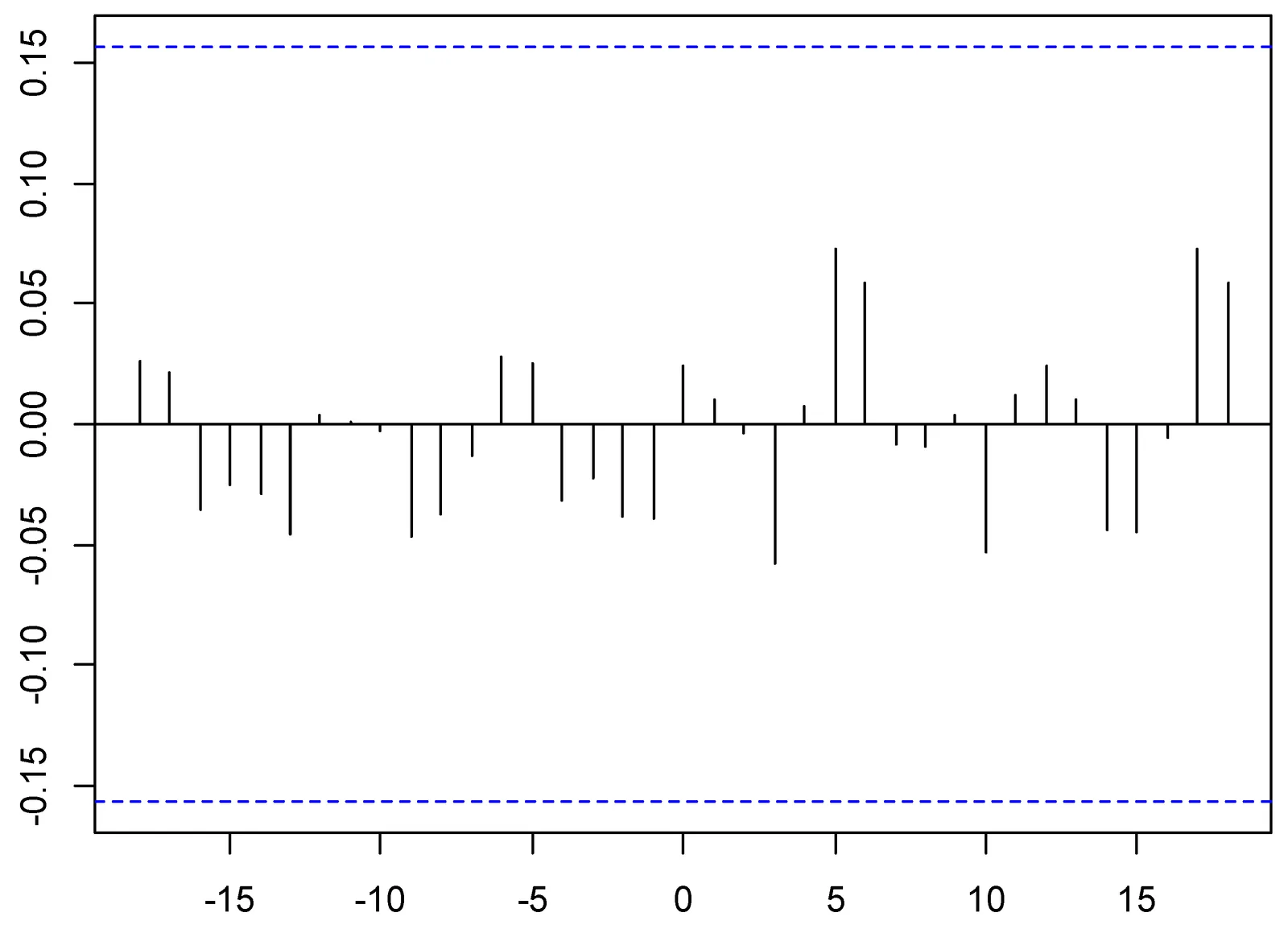
Cross-correlation function (CCF) between time series representing Eid al-Adha month and monthly number of SGP outbreaks in China in 2013–2024.

Furthermore, data from Greece showed no statistically significant cross-correlation. The negative lag values, while present, were not significant, and therefore do not support the hypothesis that SGP outbreak peaks lead the Eid al-Adha celebration month (Figure 11). Conversely, no statistically significant cross-correlation was identified between the analyzed time series at any lag values (*p* > 0.05) in China during the period 2010–2024 (Figure 12). This finding contrasts with the patterns observed in Russia, Bulgaria, Mongolia and Tajikistan, suggesting that the Eid al-Adha-related animal movements may be effectively mitigated in China by the country’s stringent veterinary control measures and centralized quarantine protocols.

### 3.4. Phylogenetic and Phylogeographic Analysis

**Figure 13:**
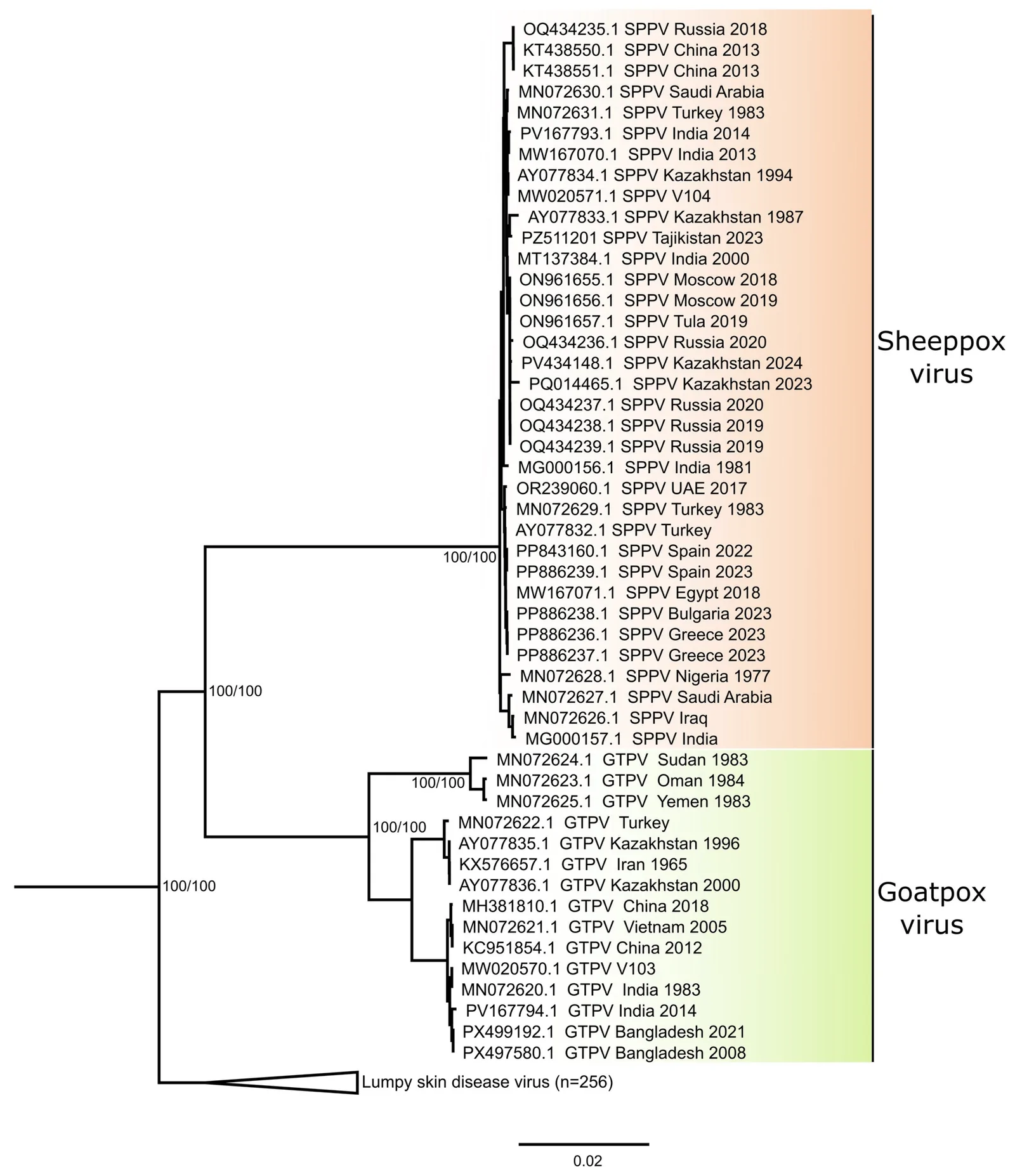
Maximum likelihood phylogeny of capripoxviruses based on complete and nearly complete genome sequences. Phylogenetic analysis was performed using IQ-TREE 3. The best-fit model of sequence evolution was selected based on the Bayesian information criterion (BIC) score [33] calculated by ModelFinder [34]. Node support was estimated by ultrafast bootstrap [35] and the SH-aLRT test [36] with 1000 replicates each. Support values are displayed at the nodes (SSH-aLRT/Ultrafast bootstrap). The tree was rooted at the midpoint. Scale bars represent the estimated average number of substitutions per site.

Phylogenetic analysis recovered three strongly supported monophyletic groups corresponding to the three recognized capripoxviruses as follows: GTPV, SPPV and LSDV (Figure 13). The GTPV clade exhibited more pronounced phylogenetic structure, characterized by deeper divergences, in comparison to the SPPV clade. Within the GTPV group, the following three distinct lineages were identified: the first comprising three genome sequences from Yemen, Oman and Sudan from the 1980s; the second lineage including four sequences from Turkey, Kazakhstan, and Iran; and the third lineage comprising GTPV genome records from Bangladesh, India, China and Vietnam. Furthermore, within the SPPV clade, phylogenetic clustering corresponded closely with the phylogenetic structure from the Sprygin et al. 2023 study [45].

**Figure 14:**
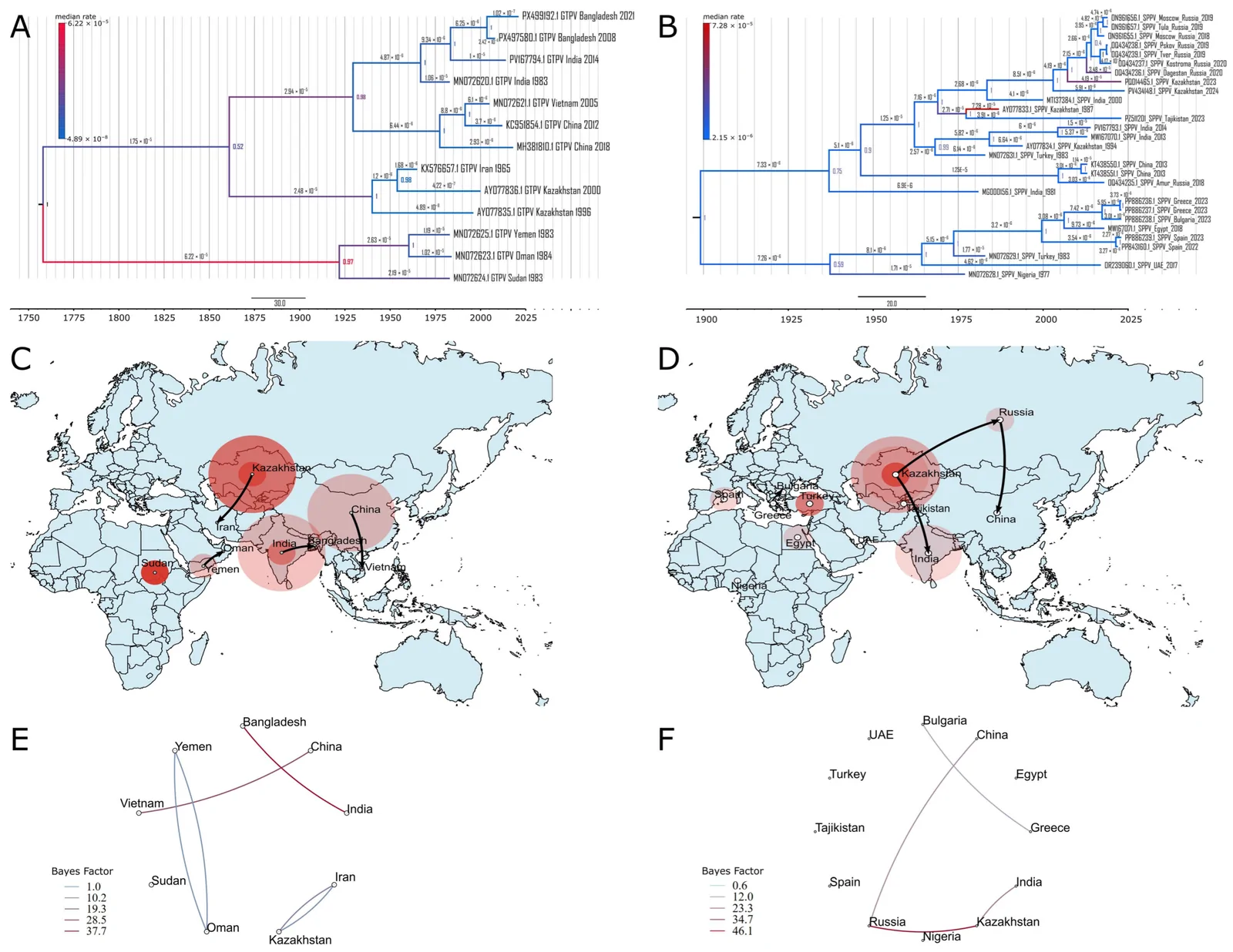
Phylodynamic and phylogeographic reconstruction of GTPV and SPPV. (**A**,**B**)—time-calibrated phylogenies of GTPV and SPPV, respectively. Branches are colored according to the estimated median substitution rate along each branch, and median values are indicated above the branches. Posterior probability support values are indicated at the nodes. (**C**,**D**)—phylogeographic reconstruction of GTPV and SPPV spread. Arrows indicate the directions of the inferred spread events. Only transmission events with Bayes factor support (BF) > 5 are shown. (**E**,**F**)—Bayes factor (BF) support of the inferred spread events between countries for GTPV and SPPV, respectively. Only transmissions with BF > 5 are shown in the circular diagrams.

Topology of the GTPV and SPPV time-calibrated phylogenies (Figure 14A,B) was largely consistent with the arrangement of the taxa in the genus-level maximum likelihood tree (Figure 13). For both, GTPV and SPPV, the median evolutionary rates were overall low, which is characteristic of DNA viruses; however, a considerable degree of rate variation across the branches was observed in both trees. Elevated rates were estimated along the deeper branches of the GTPV phylogeny (1.7–6.2 × 10^−5^ substitutions/site/year) compared to the rates closer to the tips of the tree (2.4 × 10^−6^–4.9 × 10^−8^), which could be caused by sparse sampling and low sample size (Figure 14A).

Based on the earliest available sequencing records and metadata, GTPV first emerged in the Middle East, from which it was inferred to spread from Sudan to Kazakhstan and Yemen. Subsequently, the virus spread from Kazakhstan to India and Iran and from Yemen to Oman. From India, GTPV spread to Bangladesh and China, and later from China to Vietnam (Figure 14C). The inferred transmission pathways from China to Vietnam and from India to Bangladesh demonstrated robust Bayes factor support (BF > 20), while the direction of spread between Yemen–Oman and Kazakhstan–Iran were less nearly equally supported, indicating uncertainty in the direction of spread (Figure 14E).

The phylogeographic reconstruction of the SPPV transmission suggested the initial spread from Kazakhstan to Turkey, India and Russia. From Russia, SPPV reached China. The subsequent spread of SPPV to countries in Europe, Africa, and Middle East was inferred with low confidence as a result of sparse sampling of complete/nearly complete genomes (Figure 14D). Of the inferred SPPV transmission pathways, the routes between Kazakhstan and Russia, Kazakhstan and India, as well as between Russia and China, had the strongest Bayes factor support (BF > 5) (Figure 14F). Unfortunately, the transmission pathway to Spain was not able to be determined due to lack of sampling of complete/nearly complete genomes.

### 3.5. Recombination Analysis

**Figure 15:**
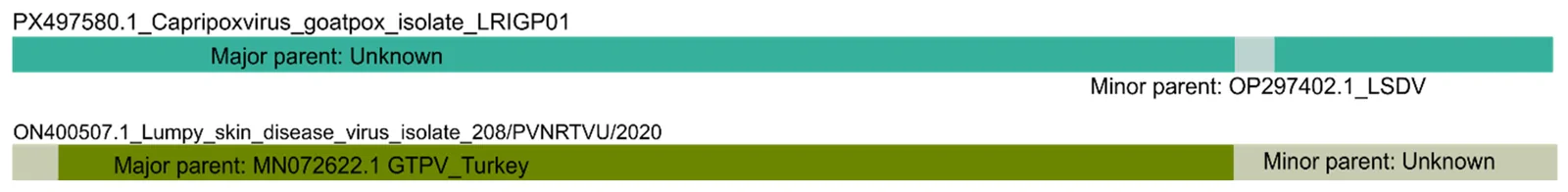
Potential recombination events involving GPV detected in the complete capripoxvirus sequence dataset. The horizontal bars represent genomic sequence; sequence names above the bar are the exemplar names of the recombinant sequences; exemplar names of the parental sequences are indicated inside or below the bar. Comprehensive details concerning detected plausible recombination events are provided in the Supplementary Table S1.

No intra-specific recombination events were detected between the SPPV and GTPV genomes. The inter-specific capripoxvirus recombination analysis identified a single recombination event in goatpox viruses, which was detected in all 15 sequences. The minor parent was identified as a lumpy skin disease virus, and the major parent was unknown (Figure 15). The recombination breakpoints were estimated to be roughly at the ends of the LRIGP 143 gene, encoding phospholipase-D-like protein. The unknown major parental sequence in the GTPV recombination pattern likely indicates a missing genomic representative in the dataset because of the scarce genome-level sequence records available for the virus. Moreover, GTPV were identified as contributing to recombination detected in lumpy skin disease viruses (Figure 15). Overall, the low number of detected recombination events highlights the importance of expanding genomic databases of SGPV. Complete information about detected plausible recombination events is provided in the Supplementary Table S1.

## 4. Discussion

### 4.1. Anthropogenic Livestock Trade as a Transboundary Spread Risk and Spatial–Temporal Drivers of SGP Endemicity

SGP represents a substantial economic threat to agricultural sectors, particularly in many areas of Africa, the Middle East, and Asia, where they are endemic. These pose a severe risk to small-scale farmers and rural communities, reducing production and causing substantial livelihood instability due to high morbidity and mortality rates [7,46]. The increased risk of infectious disease introduction and spread in both human and animal populations is a well-documented consequence of trade and migration processes. SGP follows this established pattern [47], primarily driven by the traditional seasonal transhumance of flocks in search of new pasture lands and the illegal movement of sheep and goats within the country [48], as evidenced in Turkey [49,50]. Illegal livestock movement and the ensuing expenses associated with disease eradication in countries with developed sheep farming sectors are estimated to hundreds of millions of dollars [51]. Furthermore, SPPV outbreaks in Greece (1988–2000) and Bulgaria (1995–1996) were attributable to illegal imports from Turkey [52], and the genetic relationship between the SPPV virus in Greece and Turkey as these viruses are in a distinct phylogenetic cluster.

Current epidemiological data clearly demonstrate that SGP is endemic in areas with high sheep and goat density [7,53,54]; however, underreporting of these diseases constitutes a major limitation in this study. As illustrated in Figure 1, the conducted analysis lacks data from most African, Central, and south Asian countries, where sheep and goats constitute a significant portion of the total animal population.

The observed pattern of predominant outbreak association with warm and moderate climate croplands and relatively low small ruminant densities may reflect the fact of infection of low-biosecurity herds at open pastures [55,56]. Given the contagious nature of SGP and the consequent role of fomites and contaminated pasture elements, biosecurity is important for capripoxvirus control and prevention [22].

Analysis of the potential correlations between the SGP outbreaks and the celebration of the Islamic holiday of Eid al-Adha in the Russian Federation and several other countries with similar outbreak recording periods suggests the existence of such a relationship, at least for Russia, Bulgaria, Mongolia and Tajikistan. In these countries, the month of Eid al-Adha precedes the peak in SGP outbreak reporting by one to four months. This pattern can be explained by a significant increase in animal movements from SGP-endemic regions/countries for ritual sacrifices during the celebration. The existence of a similar correlation for a range of infectious diseases is supported by the study of Beverelli and Ticku (2023) [29] and is indirectly acknowledged by a number of official statements from regional veterinary services warning the public against importing ritual animals from potentially affected regions [57]. In contrast, no such relationship was observed for countries such as Greece and China.

These findings highlight that Eid al-Adha may be considered a significant anthropogenic risk factor shaping the annual dynamics of the SGP epizootic process in countries with well-developed systems for importing small ruminants. The absence of this association in China and Greece necessitates further investigation. In the case of China, the lack of a significant cross-correlation may be attributed to the country’s highly regulated livestock trade system, which includes mandatory quarantine periods for inter-provincial animal movements, strict biosecurity protocols at the farm level, and centralized oversight of live animal markets. Unlike in Russia or Bulgaria, where seasonal cross-border movements for religious festivities are less tightly controlled, China’s comprehensive veterinary regulatory framework likely reduces the probability that festival-related imports will translate into measurable outbreak peaks. Furthermore, the relatively low proportion of small ruminants traded specifically for Eid al-Adha in China, combined with the predominance of domestic production over imports, may dampen the epidemiological impact of the holiday. In Greece, the absence of correlation could be related to different livestock marketing structures or to the fact that SGP outbreaks in recent years have been largely confined to specific regions with limited animal inflow during the festival. Nevertheless, these hypotheses remain speculative and warrant targeted epidemiological investigations with granular data on animal movements.

Outbreaks of SPPV in the Central Federal District of the Russian Federation atypically occur in regions with lower population density, indicating SPPV can spread in low density populations [45]. Movement of infected animals for Eid al-Adha is an important factor in the spread of infection in the central region of Russia (Figure 4). This epizootological conclusion is reflected in the phylogenetic data: all isolates isolated in the Central and North Caucasian Federal Districts form a single genetic cluster with isolates from Kazakhstan. Such genetic homogeneity over a wide area is consistent with the hypothesis of intensive movement of animals and virus between adjacent regions, supporting the circulation of a single SPPV. Notably, the movement of sheep to the site of the Eid al-Adha celebrations is accompanied by an increase in the incidence of SPPV outbreaks in Turkey [58].

Epidemiological surveillance and field studies identified a robust temporal correlation between religious festivities—specifically Eid al-Adha—and a heightened incidence of SSPV outbreaks. During these periods, the convergence of high-density animal aggregations and the intensive, often unregulated, movement of small ruminants across open markets facilitates rapid viral transmission. Research indicates that the absence of veterinary inspection, testing, and quarantine during the pre-holiday transportation phase serves as a primary driver for the emergence of new infection clusters [59,60]. Spatial and temporal analyses further characterize points of local livestock sales as critical hotspots for viral dissemination; statistical modeling indicates that surges in meat demand during these peak seasonal windows can result in a 35% increase in reported outbreaks within rural vicinities. This trend is largely substantiated by the influx of animals from endemic regions, circumvention of official health controls, and a widespread failure to adhere to mandatory quarantine periods prior to market introduction [61,62]. Furthermore, cases of contagious ecthyma, characterized by pustular lesions on the skin of the hands, are often recorded in humans when in contact with infected animals during these holidays [63,64].

### 4.2. Capripoxvirus Evolution and Recombination Dynamics

The use of WGS has led to significant advancements in understanding viral spread. The phylogeny of capripoxviruses has garnered heightened interest due to geographic expansion [65]. Furthermore, sequence information, together with epidemiological data concerning outbreaks, provides the resolution necessary to identify a complete picture of genetic variation across CaPVs [66,67].

Sanger sequencing was historically the primary tool for reconstructing the phylogenetic analysis of pathogens. In the study of capripoxviruses, the RPO30 and GPCR genes were initially proposed as diagnostic markers to differentiate among SPPV, GTPV, and LSDV [68,69,70]. However, these single-locus approaches lack the necessary resolution to identify the genetic variation within the genus. Due to the inherently low mutation rates characteristic of capripoxviruses, the genetic signal provided by a single gene is often insufficient for robust phylogenetic inference or the fine-scale discrimination of closely related lineages [71].

Despite the substantial number of small ruminants raised in the Eurasian region, findings from this area remain scarce. Phylogenetic analyses based on the whole-genome sequences of the SPPV genomes from Russia demonstrated that the studied isolates from the Central and North Caucasus Federal Districts exhibit significant genetic divergence from the attenuated NISKHI vaccine strain. It is imperative to acknowledge that the attenuated NISKHI vaccine strain was originally obtained through successive passages of a locally isolated virulent strain that was circulating in Kazakhstan [72]. All the SPPV outbreaks within the Russian Federation are not associated with the NISKHI vaccine strain, thereby confirming its safety for utilization in susceptible animal population.

A knowledge gap persists regarding the molecular epidemiology of SPPV in central Asian countries, primarily driven by a lack of complete genome sequences. However, a preceding study on the outbreaks of SPPV in the Russian Far East identified SPPV similar to SPPV from China and India [73]. Furthermore, sequence data based on a partial region of the P32 gene demonstrated that the SPPV field isolates from Mongolia were identical to the corresponding gene sequences of the SPPV isolates from India, as well as a SPPV A strain from Kazakhstan [14].

The data indicate the presence of two distinct genetic lineages of the SPPV widespread within the Russian Federation. The isolates from the Central and North Caucasian Federal Districts are clustered into one group, and the strain from Amur (Amur/2018) is clustered with a sequence of SPPV from China. It is also noteworthy that the isolates from Kazakhstan in 2023–2024, including the VOAO50-Oil-2023 strain, are closely clustered with a group of SPPV isolated in the Central Federal District and North Caucasus Federal District of Russia, indicating that the SPPV is circulating in the surrounding regions, represented by a cluster of isolates unique to the region.

Interestingly, the Tajik SPPV 2024 exhibited a genetic similarity to the SPPV A from Kazakhstan dated 1994, which lends support to the existence of the unique region-specific genetic cluster (Figure 12).

During the 2022–2023 period, Spain, Bulgaria, and Greece reported occurrences of SPPV outbreaks, which, following WGS, were grouped together with the SPPV causing the recent European outbreaks and those currently circulating in Egypt and Turkey, indicating the presence of an additional genetic pool (Figure 12).

Given the high degree of overall similarity between the available vaccine and field strains, the phylogenetic position of a strain cannot guide assumptions regarding the phenotypic manifestation [74]. Further analysis of specific genes involved in virulence may be useful for determination of virulence [75].

### 4.3. Regional Veterinary Control Implications

Given the contagious nature of SGP and the consequent role of fomites and contaminated pasture elements, biosecurity is important for Capripoxvirus control and prevention [22].

All the SPPV outbreaks within the Russian Federation are not associated with the NISKHI vaccine strain, thereby confirming its safety for utilization in susceptible animal population.

### 4.4. Study Limitations and Future Research Directions

Underreporting of these diseases constitutes a major limitation in this study. As illustrated in Figure 1, the conducted analysis lacks data from most African, central, and south Asian countries, where sheep and goats constitute a significant portion of the total animal population.

A knowledge gap persists regarding the molecular epidemiology of SPPV in central Asia countries, primarily driven by a lack of complete genome sequences.

Further analysis of specific genes involved in virulence may be useful for determination of virulence [75].

## 5. Conclusions

This study provides a comprehensive spatiotemporal and phylogenetic characterization of sheeppox and goatpox outbreaks over the past 15 years. Our epidemiological analysis confirms that SGP outbreaks predominantly occur in small-scale holdings (<100 head), are associated with warm cropland ecosystems and moderate animal densities (10–50 head/km^2^) and exhibit a distinct seasonal peak in late summer. The significant cross-correlation between the month of Eid al-Adha and subsequent outbreak peaks in Russia, Bulgaria, Mongolia and Tajikistan highlights the role of festival-related animal movements as a major anthropogenic risk factor. In contrast, the absence of such a correlation in China suggests that stringent veterinary controls, centralized quarantine systems, and limited reliance on imported sacrificial animals may effectively mitigate this risk, though this hypothesis warrants further investigation with granular movement data.

Phylogeographic reconstructions identify key transboundary transmission routes, notably from Kazakhstan to Russia and India for SPPV, and from China to Vietnam for GTPV, with strong Bayes factor support. Recombination analysis reveals no intra-specific recombination but a single inter-specific event involving GTPV and LSDV, indicating that genetic exchange within the genus is limited but not absent. These findings underscore the value of whole-genome sequencing for high-resolution molecular surveillance and for tracing the spread of capripoxviruses across borders.

Nevertheless, our study is constrained by incomplete outbreak reporting and sparse genomic sampling from large parts of Africa and central/south Asia, which limits the generalizability of the inferred transmission pathways. Future efforts should prioritize filling these data gaps through enhanced surveillance and sequencing, as well as integrating detailed animal movement data to refine risk assessments and guide targeted control measures, particularly during high-risk periods such as religious festivals.

## Figures and Tables

**Table 1:** The epidemiological indicators of the SGP outbreaks worldwide between 2010 and 2024.

Number of the Susceptible Animals	Number of Outbreaks	Morbidity, % Mean (95%CI)	Mortality, % Mean (95%CI)
Less than 100 head	686	21.0 (18.5–23.5)	2.5 (1.8–3.2)
100–1000 head	642	6.6 (5.6–7.6)	2.0 (1.5–2.6)
More than 1000 head	43	3.1 (2.8–3.4)	1.8 (1.5–2.1)

## Data Availability

The SPPV sequence Tajikistan/2023 is deposited in Genbank under PZ511201.

## Supplementary Materials

The following supporting information can be downloaded at: https://www.mdpi.com/article/10.3390/v18080849/s1, Supplementary Table S1. Recombination.
